# Tamoxifen induces radioresistance through NRF2-mediated metabolic reprogramming in breast cancer

**DOI:** 10.1186/s40170-023-00304-4

**Published:** 2023-02-08

**Authors:** F. V. Reinema, F. C. G. J. Sweep, G. J. Adema, W. J. M. Peeters, J. W. M. Martens, J. Bussink, P. N. Span

**Affiliations:** 1 Department of Radiation Oncology, Radboud University Medical Center, Nijmegen, 6500 HB the Netherlands; 2 Department of Laboratory Medicine, Radboud University Medical Center, Nijmegen, The Netherlands; 3 Department of Medical Oncology, Erasmus University Medical Centre, Rotterdam, The Netherlands

**Keywords:** Tamoxifen, Radiotherapy, Breast cancer, Reactive oxygen species, Antioxidants, NRF2

## Abstract

**Background:**

Recently, we reported that tamoxifen-resistant (TAM-R) breast cancer cells are cross-resistant to irradiation. Here, we investigated the mechanisms associated with tamoxifen-induced radioresistance, aiming to prevent or reverse resistance and improve breast cancer treatment.

**Methods:**

Wild-type ERα-positive MCF7 and ERα-negative MDA-MB-231 breast cancer cells and their TAM-R counterparts were analyzed for cellular metabolism using the Seahorse metabolic analyzer. Real-time ROS production, toxicity, and antioxidant capacity in response to H_2_O_2_, tamoxifen, and irradiation were determined. Tumor material from 28 breast cancer patients before and after short-term presurgical tamoxifen (ClinicalTrials.gov Identifier: NCT00738777, August 19, 2008) and cellular material was analyzed for NRF2 gene expression and immunohistochemistry. Re-sensitization of TAM-R cells to irradiation was established using pharmacological inhibition.

**Results:**

TAM-R cells exhibited decreased oxygen consumption and increased glycolysis, suggesting mitochondrial dysfunction. However, this did not explain radioresistance, as cells without mitochondria (Rho-0) were actually more radiosensitive. Real-time measurement of ROS after tamoxifen and H_2_O_2_ exposure indicated lower ROS levels and toxicity in TAM-R cells. Consistently, higher antioxidant levels were found in TAM-R cells, providing protection from irradiation-induced ROS. NRF2, a main activator of the antioxidant response, was increased in TAM-R cells and in tumor tissue of patients treated with short-term presurgical tamoxifen. NRF2 inhibition re-sensitized TAM-R cells to irradiation.

**Conclusion:**

Mechanisms underlying tamoxifen-induced radioresistance are linked to cellular adaptations to persistently increased ROS levels, leading to cells with chronically upregulated antioxidant capacity and glycolysis. Pharmacological inhibition of antioxidant responses re-sensitizes breast cancer cells to irradiation.

**Supplementary Information:**

The online version contains supplementary material available at 10.1186/s40170-023-00304-4.

## Background

Breast cancer is the most prevalent cancer in female adults and is generally treated with a combination of surgery, radiotherapy, and chemotherapy. Additionally, endocrine treatment is an important therapy for the 80% of breast cancers expressing estrogen receptor alpha (ERα) [1]. A frequently used endocrine therapy agent is tamoxifen, which is extensively used in the adjuvant and metastatic setting. As a selective estrogen receptor modulator, tamoxifen inhibits estrogen-mediated proliferation [2–4] in ERα-positive breast cancer cells. Despite a good response rate, around 40% of patients treated with tamoxifen develop treatment resistance and recurrent disease, decreasing survival [5].

Recently, we reported that tamoxifen-resistant (TAM-R) breast cancer cells are cross-resistant to radiotherapy [6]. As radiotherapy is essential in both primary treatment and palliative care, this resistance, if translated to the clinical setting, might strongly decrease treatment options for patients relapsing after tamoxifen treatment. Our previous research has related tamoxifen- and radioresistance to upregulated interferon signaling pathways [6]. However, we were unable to establish a causal relationship between interferon signaling and irradiation sensitivity.

Radiotherapy causes tumor cell death through its ionizing activity with the consequential formation of free radicals as reactive oxygen species (ROS), eventually causing irreparable DNA double strand breaks [7]. Resistance to radiotherapy can be acquired through for instance mitochondrial dysfunction [8, 9], increased glycolysis, and other metabolic adaptations [10]. Interestingly, besides pharmacologically inhibiting the ERα, tamoxifen acts as an inhibitor of mitochondrial oxidative phosphorylation (OXPHOS) complexes 1 and 3 [11, 12]. These latter effects are ERα-independent and result in cytotoxicity through inhibition of mitochondrial bioenergetics and increased cellular ROS levels [13]. We therefore hypothesized that mitochondrial dysfunction and/or metabolic rewiring after prolonged tamoxifen treatment may be associated with subsequent radioresistance.

Our data using chronic tamoxifen treatment in ERα-positive and -negative breast cancer cell lines revealed that TAM-R cells exhibit a more glycolytic phenotype than WT cells. They produce less ROS and are better protected against oxidative damage through higher antioxidant capacity. Moreover, tumors of breast cancer patients treated with tamoxifen before surgery, exhibit increased antioxidant expression (NCT00738777). Pharmacological inhibition of the antioxidant response re-sensitized treatment-resistant breast cancer cells to irradiation.

## Methods

### Cell culture

MCF7 breast cancer cells (LGC Standards) were cultured in DMEM glutamax (Gibco) supplemented with 10% fetal bovine serum, human insulin (6 μg/ml, Sigma) and penicillin/streptomycin (10 U/ml, Gibco) at 37 °C, 5% CO2. MDA-MB-231 cells (LGC Standards) were cultured in DMEM glutamax (Gibco) supplemented with 10% fetal bovine serum, sodium pyruvate (1 × , Gibco) and penicillin/streptomycin. 67NR cells (kindly provided by Mirjam Zegers, Department of Cell Biology, Radboudumc Nijmegen) were cultured in RPMI (Gibco) supplemented with 10% fetal bovine serum, sodium pyruvate, penicillin/streptomycin, ultra-glutamine (2 mM, Lonza), and nonessential amino acids (1 × , Gibco).

All experiments with tamoxifen were performed using the active metabolite 4-hydroxy-tamoxifen (H7904, Sigma). MCF7 and MDA-MB-231 cells were cultured tamoxifen-resistant (MCF7^TAM−R^ and MDA-MB-231^TAM−R^) by chronically culturing cells with increasing doses of tamoxifen. After obtaining resistance, MCF7^TAM−R^ were resistant to 5 μM, whereas MDA-MB-231^TAM−R^ cells could be maintained with 10 μM tamoxifen reflecting clinically relevant concentrations.

### Colony-forming assays

For colony-forming assays, cells were plated in 6-wells cell culture plates (Corning) and incubated 24 h for adherence before single dose irradiation with 1, 2, 4, 6, and 8 Gy (X-RAD Biological Irradiator; Precision X-ray). Cells were fixed and stained with crystal violet. Colonies were counted manually, and surviving fractions calculated.

### ROS measurement

ROS were fluorescently detected with CellROX™ Green (10 μM, C10444, Thermo Fisher) on the Incucyte ZOOM (Essen Bioscience). Cells were seeded in 96 wells cell culture plates and incubated to adhere. CellROX was added 1 h after treatment with tamoxifen (1, 2.5, 5, and 10 μM) or OXPHOS inhibitors Metformin (0.03, 2, 5, 10 mM, Sigma) and IACS-010759 (0.1, 0.3, 1, and 3 μM, Selleckchem). Tert-butyl hydroperoxide (200 μM; Sigma) was used as positive control. ROS fluorescence was followed for 24 h by automated live cell imaging. Area under the curve was calculated for statistical analyses.

### Toxicity measurement

Toxicity of H_2_O_2_ on breast cancer cells were measured with the CellTOX™ Green cytotoxicity assay (G8741, Promega) on the Incucyte ZOOM (Essen Bioscience). Cells were seeded and incubated in 96 wells cell culture plates to adhere. CellTOX was added to the cells 1 h prior to adding H_2_O_2_ (200 μM) to assess basal toxicity. CellTOX fluorescence was followed for 48 h by automated imaging. Area under the curve was calculated for statistical analyses.

### Metabolism

Real-time metabolic assessment of cellular mitochondrial function was performed using the Seahorse XF-96 Extracellular Flux Analyzer (Agilent), measuring oxygen consumption and lactate production with several mitochondrial respiratory chain inhibitors. Cells were seeded in Seahorse XF-96 microplates (Agilent) and incubated to attach overnight. Medium was replaced with Seahorse assay medium (8.3 g DMEM powder, 0.016 g phenol red and 1.85 g NaCl in 1 L Milli-Q water, pH 7.4 supplemented with 11 mM glucose, 2 mM L-glutamine and 1 mM pyruvate) with different concentrations of tamoxifen (1, 5, 10 μM) or vehicle and incubated 1 h (37 °C, 0% CO2) prior to measurement. For the Mito Stress Test, oligomycin A (1 μM), FCCP (carbonyl cyanide-p-trifluoromethoxyphenylhydrazone, 1 μM), and antimycin A (2.5 μM) with rotenone (1.3 μM) were used, whereas for the Glyco Stress Test, glucose (11 mM), oligomycin A (1 μM), and 2-deoxy-D-glucose (22 mM) were injected during measurement.

Wave 2.3.0 was used for data analysis. Spare respiratory capacity (SRC) was calculated as the difference between basal oxygen consumption rate (OCR) and maximal OCR as measured under FCCP.

### Rho0 culture

MDA-MB-231 and 67NR cells were mitochondrial depleted by chronic treatment with up to 100 ng/ml Ethidium Bromide (Sigma), a method first used in yeast cells to deplete cells from mitochondrial DNA [14]. Medium as described above was additionally supplemented with 50 μg/ml uridine (Sigma), and 100 μg/ml sodium pyruvate (Gibco).

### Mitochondrial staining

Mitochondrial depletion was confirmed by live staining with MitoTracker™ Orange CMTMRos (100 nM, M7510, Thermo Fisher) for 30 min before fixing cells in 4% PFA. Hoechst 33,342 (Sigma) was applied for nuclear staining.

### Antioxidant concentration

Antioxidant capacity was measured in cell lysates with the Antioxidant assay kit (CS0790, Sigma).

### Gene expression patients

Tumor gene expression was obtained from breast cancer patients enrolled in a pre-operative trial (ClinicalTrials.gov Identifier: NCT00738777). Patients provided informed consent for participation in the study. The aim of the study was to prospectively investigate molecular changes induced by short-term endocrine treatment. Study details and patient characteristics can be found in a previous publication [15]. After biopsies were taken and paraffine embedded, premenopausal patients received 40 mg tamoxifen trice daily for the first week, followed by 20 mg daily for the remaining treatment period. Postmenopausal patients were randomized between tamoxifen according to the schedule mentioned above, or neoadjuvant anastrozole (1 mg daily ± fulvestrant, 500 mg day (d) 0, d15, d29, and once every 28 days thereafter until surgery). Treatment was scheduled for 3 weeks (± 1 week) after which tumors were surgically removed, embedded in paraffine, and analyzed for gene expression.

Gene expression data from the above-mentioned trial is available for the patients treated with short-term presurgical tamoxifen in GEO (Platform ID: GPL28292, Series GSE147271). Twenty-eight patients for whom paired data were available from before and after treatment were selected. Tumor gene expression of NFE2L2 was analyzed for the two groups *Pretreatment* and *Tamoxifen* with the *R*-script available in GEO.

### NRF2 staining

Tumor material was obtained from 12 breast cancer patients who were enrolled in the above-mentioned pre-operative trial at Radboudumc, Nijmegen. Tumor sections were stained for NRF2 by hematoxylin staining with an anti-NRF2 antibody (D1Z9C, Cell Signaling Technology; dilution 1/50) and secondary antibody BrightVision Goat anti rabbit IgG, HRP labelled (VWRKDPVO110HRP, ImmunoLogic). Sections were scored blinded and evaluated for consensus. microscopic images were obtained on a Leica DM6000 microscope (Leica).

Cells were seeded in chamber slides (Lab-Tek, Thermo Fisher) and fixed in paraformaldehyde (4%), 28 h after seeding. After permeabilization (0.1% Triton-X, Sigma), and blocking, primary antibody against NRF2 (D1Z9C, Cell Signaling, dilution 1/800) was applied 45 min at room temperature. Anti-rabbit-488 (A21206, Invitrogen, dilution 1/300) was used as secondary antibody. Cells were counterstained with Hoechst. For each cell line, 6 images were taken randomly at a Zeiss LSM900 microscope and nuclear foci were quantified using Fiji/ImageJ.

### RNA sequencing

RNA-sequencing results were used as obtained in Post et al. [6]. Fold change in expression levels of MCF7^TAM−R^ compared to MCF7^WT^ of normalized read counts was reanalyzed for genes related to the antioxidant function of NRF2 as reported in literature [16–19].

### Antioxidant inhibition

ML385 (5 μM; Selleckchem) in DMSO (final concentration 0.01%) was added to the cells 24 h prior to assessing toxicity to H_2_O_2_ (200 μM) or irradiating for colony formation assays as described above.

### Statistical analysis

Statistical analysis was performed using GraphPad Prism 8.01 software with two-sided Student’s *t* test after normality was confirmed, or ANOVA with Sidak post hoc test where applicable. Specifically, to analyze clonogenic survival for radiotherapy response, a linear–quadratic model for curve fitting was used. ROS signal intensity was analyzed by Student’s *t* test with Bonferroni correction for multiple testing on area under the curve comparing each condition between WT and TAM-R cells. Tumor gene expression of 28 patients was statistically analyzed with a paired *t* test in *R* software. Fold change in RNA gene expression levels was analyzed by one sample Wilcoxon test after normality was disproven. Data is generally shown as mean ± SD or SEM. The number of biological and technical replicates are shown in the figure legends. A *p* value < 0.05 was considered statically significant.

### Data availability

The data generated in this study are available upon request from the corresponding author. The gene expression data analyzed in this study were obtained from Gene Expression Omnibus (GEO) at GSE147271.

## Results

### Long-term tamoxifen treatment renders breast cancer cells resistant to irradiation irrespective of ER status

**Fig. 1:**
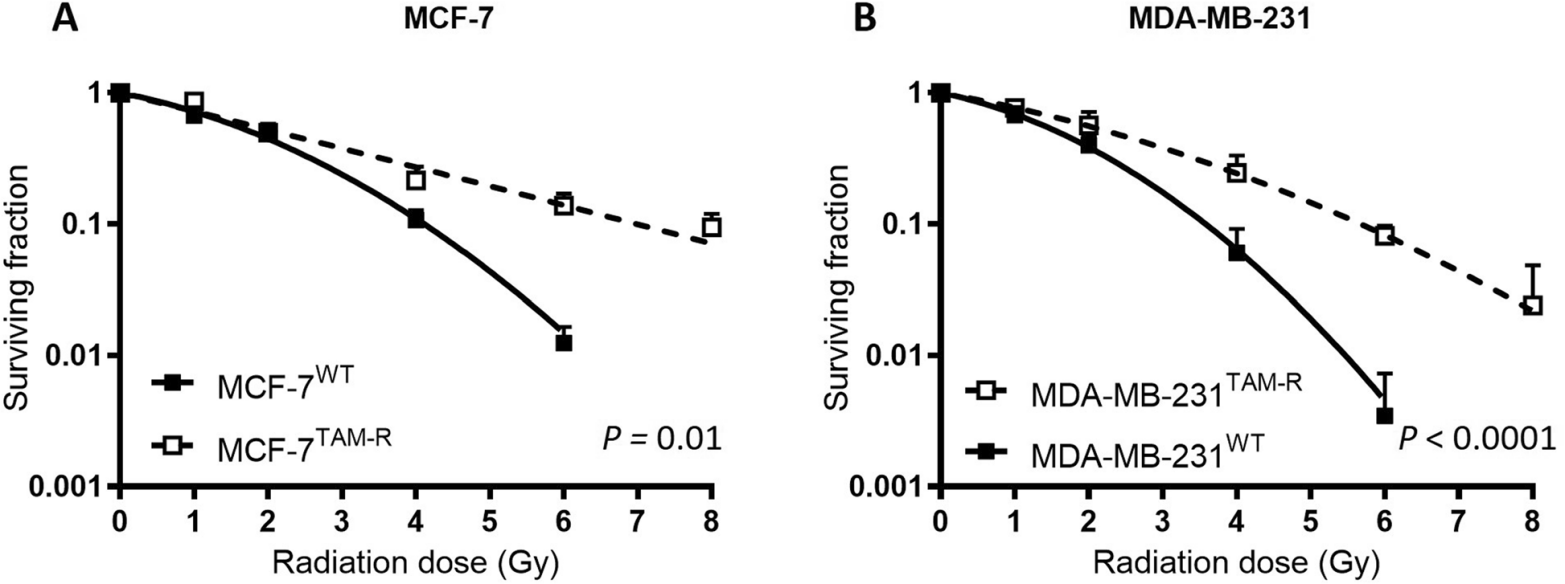
Resistance for irradiation after long-term tamoxifen treatment in MCF7 and MDA-MB-231 cells. **A** Clonogenic survival assay of MCF7^WT^ and MCF7^TAM−R^ after irradiation. **B** Clonogenic survival assay of MDA-MB-231^WT^ and MDA-MB-231^TAM−R^ after irradiation. Data are represented as mean ± SD. Data are represented as mean + SD (*n* = 6), and statistical significance was determined by comparison of fitted curves

MCF7 and MDA-MB-231 breast cancer cells that were chronically cultured with tamoxifen (MCF7^TAM−R^ and MDA-MB-231^TAM−R^) were assessed for their response to irradiation in clonogenic survival assays. Both MCF7^TAM−R^ and MDA-MB-231^TAM−R^ survived higher doses of irradiation than wild-type (WT) cells (Fig. 1). Confirming earlier data [6], the surviving fraction was significantly increased in MCF7^TAM−R^ compared to MCF7^WT^ (*p* = 0.01; Fig. 1A). Also, ERα-negative breast cancer cells MDA-MB-231^TAM−R^ exhibited significantly higher surviving fractions after irradiation than MDA-MB-231^WT^ (*p* < 0.0001; Fig. 1B). While the WT cells’ survival was < 0.001 after 8 Gy, their TAM-R counterparts did form colonies after treatment at this dose.

### Tamoxifen-resistant cells are more glycolytic than wild-type cells

After prolonged tamoxifen treatment, metabolic adaptations may explain the cross-resistance to irradiation in cancer cells, as tamoxifen reportedly acts as an inhibitor of OXPHOS complexes 1 and 3 [11, 12] while increased glycolysis is known to play a role in the radiosensitivity of cells [10]. We assessed whether acute or prolonged tamoxifen treatment would change cellular metabolism including OXPHOS and glycolysis. Using the Seahorse extracellular flux analyzer, we measured oxygen consumption rate (OCR) and extracellular acidification rate (ECAR) over time in a live cell metabolic assay.

**Fig. 2:**
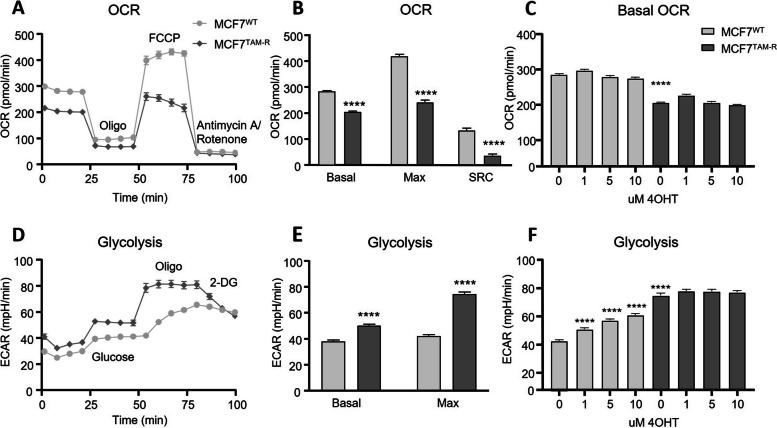
Differences in OCR and ECAR between MCF7^WT^ and MCF7^TAM−R^. **A** Mito stress test of MCF7^WT^ and MCF7^TAM−R^ cells with oligomycin (Oligo), FCCP and antimycin A/rotenone shows a decreased OCR for MCF7^TAM−R^. **B** Basal, maximal (Max) and spare respiratory capacity (SRC) are decreased in MCF7^TAM−R^ cells. **C** Basal OCR with increasing doses of tamoxifen. **D** Glyco stress test of MCF7^WT^ and MCF7^TAM−R^ cells with glucose, oligomycin (Oligo), and 2-DG shows an increased ECAR for MCF7^TAM−R^. **E** Basal and maximal (Max) ECAR are increased in MCF7^TAM−R^ cells. **F** Maximal ECAR with increasing concentrations of 4OHT in MCF7^WT^ and MCF7.^TAM−R^ cells. Data are represented as mean ± SEM (**A**, **D**) and mean + SEM (**B**, **C**, **E**, **F**; *n* = 5). Statistical significance was determined by Student’s *t* test comparing WT with TAM-R (**B**, **E**) and with ANOVA with Sidak post hoc test (**C**, **F**). ****p < 0.0001

MCF7^TAM−R^ exhibited a decreased OCR compared to MCF7^WT^ (Fig. 2A). The basal OCR (*p* < 0.0001) as well as the maximal OCR as measured after FCCP injection (*p* < 0.0001) were significantly decreased in TAM-R cells. Following, MCF7^TAM−R^ also had a significantly lower spare respiratory capacity (SRC; *p* < 0.0001; Fig. 2B). On the other hand, acute tamoxifen treatment, 1 h prior to measurement with different doses (1, 5 and 10 uM) did not affect the basal OCR in WT or TAM-R MCF7 cells (Fig. 2C).

Glycolysis, as a major energy pathway related to radioresistance [10], was measured by glyco-stress test. This assay depends on the conversion of glucose to lactate leading to an acidification of the medium and was measured as extracellular acidification rate (ECAR) in the Seahorse system (Fig. 2D). The maximal glycolytic capacity induced by oligomycin was reached much slower in MCF7^WT^ than in MCF7^TAM−R^. During the 25 min of measurement, the maximal ECAR in MCF7^WT^ cells kept increasing, while in the MCF7^TAM−R^ cells, the maximal glycolytic capacity was reached immediately after injection of oligomycin. The basal ECAR measured after glucose injection as well as the maximal glycolytic capacity were significantly increased (both *p* < 0.0001) in MCF7^TAM−R^ compared to MCF7^WT^ (Fig. 2E).

To assess whether and at what concentration acute tamoxifen treatment has the capacity to also increase glycolysis in MCF7^WT^ cells, we incubated the cells with different concentrations 1 h before starting measurement in the Seahorse analyzer. Tamoxifen significantly increased the maximal ECAR in MCF7^WT^ in a concentration dependent manner (all *p* < 0.0001 compared to control; Fig. 2F). However, increasing doses of tamoxifen did not further influence the glycolytic capacity in MCF7^TAM−R^.

We also performed this range of experiments with the MDA-MB-231 cells, measuring OCR and glycolysis after chronic and acute tamoxifen treatment in MDA-MB-231^WT^ and MDA-MB-231^TAM−R^. However, no difference in OCR or ECAR was detected between the WT and TAM-R cells or with acute tamoxifen treatment (Supplementary Figure S1).

### Mitochondrial depletion increases cellular sensitivity to irradiation

Considering the decrease in mitochondrial respiration after prolonged tamoxifen treatment, we assessed whether mitochondrial dysfunction was a direct cause of subsequent radioresistance, mimicking mitochondrial dysfunction by mitochondrial depletion using ethidium bromide [14]. After 8 weeks, no mitochondria were detectable (Supplementary Figure S2). Mitochondrial dysfunction did not explain radioresistance as cells without mitochondria (Rho0) turned out to actually be more radiosensitive than WT cells with intact mitochondria (Supplementary Figure S2).

### Tamoxifen induces mitochondrial ROS in wild-type cells, but not in tamoxifen-resistant cells

Reportedly, mitochondrial OXPHOS inhibition leads to increased ROS production [20], which could also be the case with tamoxifen. We assessed whether tamoxifen increases ROS levels and compared differences between WT and TAM-R cells, and WT and Rho0 cells, by measuring the fluorescent intensity of CellROX indicating real-time cellular ROS production.

**Fig. 3:**
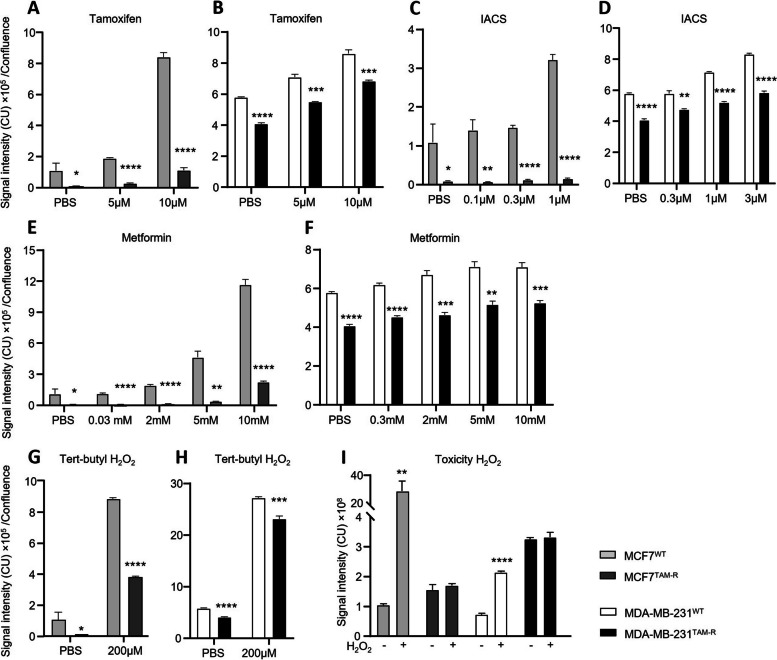
ROS production in MCF7 and MDA-MB-231 cells after treatment with different OXPHOS inhibitors and H_2_O_2_. Signal intensity of CellROX was measured after several concentrations of tamoxifen (**A**,** B**), IACS (**C**,** D**), Metformin (**E**,** F**) and Tert-butyl hydroperoxide (**G**,** H**) and was generally lower in TAM-R cells. Toxicity of H_2_O_2_ (**I**). Data are represented as mean + SD (*n* = 3). Statistical significance was determined by Student’s *t* test with Bonferroni correction for the area under the curve comparing TAM-R cells to WT cells per condition. *, *p* < 0.05; **, *p* < 0.01; ***, *p* < 0.001; ****, *p* < 0.0001

Treatment with 5 μM and 10 μM tamoxifen increased ROS levels in a dose dependent way in all cell types, but significantly lower ROS levels were observed in TAM-R cells compared to WT. Tamoxifen barely increased ROS levels in MCF7^TAM−R^ compared to MCF7^WT^ (5 μM *p* < 0.0001, 10 μM *p* < 0.0001; Fig. 3A). Also in MDA-MB-231 cells, ROS levels after tamoxifen treatment were significantly lower in MDA-MB-231^TAM−R^ compared to MDA-MB-231^WT^ (5 μM *p* = 0.0002, 10 μM *p* = 0.0006; Fig. 3B).

Presuming that tamoxifen acts as an OXPHOS inhibitor, we assessed whether this effect was similar using other OXPHOS inhibitors, and measured ROS after treatment with OXPHOS inhibitors IACS-010759 (complex 1) and Metformin (complex 3). Like tamoxifen, Metformin and IACS-010759 dose-dependently increased ROS production in MCF7^WT^, but much less in MCF7^TAM−R^. IACS-010759 increased ROS levels from 1 μM in MCF7^WT^ (Fig. 3C) and MDA-MB-231^WT^ (Fig. 3D). However, ROS levels in TAM-R cells remained significantly lower than in WT cells after IACS-010759 treatment. Concentrations higher than 2 mM of Metformin also increased ROS fluorescent intensity in MCF7^WT^ whereas we only observed a minor increase in MCF7^TAM−R^ after 10 mM Metformin (Fig. 3E). Contrary to MCF7 cells, Metformin had little effect on MDA-MB-231 cells. ROS levels in both WT and TAM-R cells only increased marginally with increasing doses of Metformin. However, ROS levels in MDA-MB-231^TAM−R^ stayed significantly lower than in MDA-MB-231^WT^ cells independently of the dose (Fig. 3F).

Strikingly, also with the positive control tert-butyl hydroperoxide, the signal intensity of CellROX was about twice as high in MCF7^WT^ compared to MCF7^TAM−R^ (*p* < 0.0001, Fig. 3G) and significantly lower in MDA-MB-231^WT^ than in MDA-MB-231^TAM−R^ (*p* = 0.0002; Fig. 3H).

To assess whether the increase in ROS measured in the WT cells originates from the mitochondria, we measured ROS production in 67NR^WT^ and mitochondrially depleted 67NR^Rho0^ cells. Just as shown for MCF7^WT^ and MDA-MB-231^WT^ cells, also 67NR^WT^ cells displayed significantly increased ROS levels after treatment with tamoxifen already after treatment with 1 μM of tamoxifen (*p* = 0.0001) which dose-dependently increased up to 10 μM tamoxifen (*p* < 0.0001; Supplementary Figure S3) which was the highest dose used for treatment. 67NR^Rho0^ cells on the other hand, displayed significantly lower ROS levels than 67NR^WT^ cells in all concentrations (*p* < 0.0001). They did not show an increase in ROS levels up to 5 μM of tamoxifen, where ROS were increased to a level comparable to treatment with 1 μM in 67NR^WT^ cells. Only with 10 μM, ROS levels were strongly increased (Supplementary Figure S3), but remained half as high as in the 67NR^WT^ cells.

### ROS are less toxic to tamoxifen-resistant cells

As we show lower ROS levels in TAM-R cells compared to WT cells not only after tamoxifen and OXPHOS inhibition, but also with tert-butyl hydroperoxide, it is likely that TAM-R cells not only produce less ROS, but are also better protected from its toxic effect by efficient radical scavenging. As the main cell-death inducing effects of radiotherapy rely upon DNA damage through ROS, better cellular ROS protection might pose an important mechanism by which cells become less sensitive to irradiation as observed after chronic tamoxifen treatment. To investigate if TAM-R cells are better protected against ROS, we assessed toxicity of H_2_O_2_ in MCF7 and MDA-MB-231 cells. H_2_O_2_ significantly increased the fluorescent intensity of CellTOX in WT cells (*p* = 0.0018 for MCF7^WT^, *p* < 0.0001 for MDA-MB-231^WT^), proving its cellular toxicity. However, there was no increase in toxicity in either MCF7^TAM−R^ or MDA-MB-231^TAM−R^ cells after H_2_O_2_ treatment (Fig. 3I). These results sustain the hypothesis that TAM-R cells have evolved more efficient radical scavenging mechanisms against ROS which could eventually protect these cells against the DNA damaging effects of radiotherapy.

### Tamoxifen increases antioxidant capacity in resistant cells and increased antioxidant gene expression in patients

**Fig. 4:**
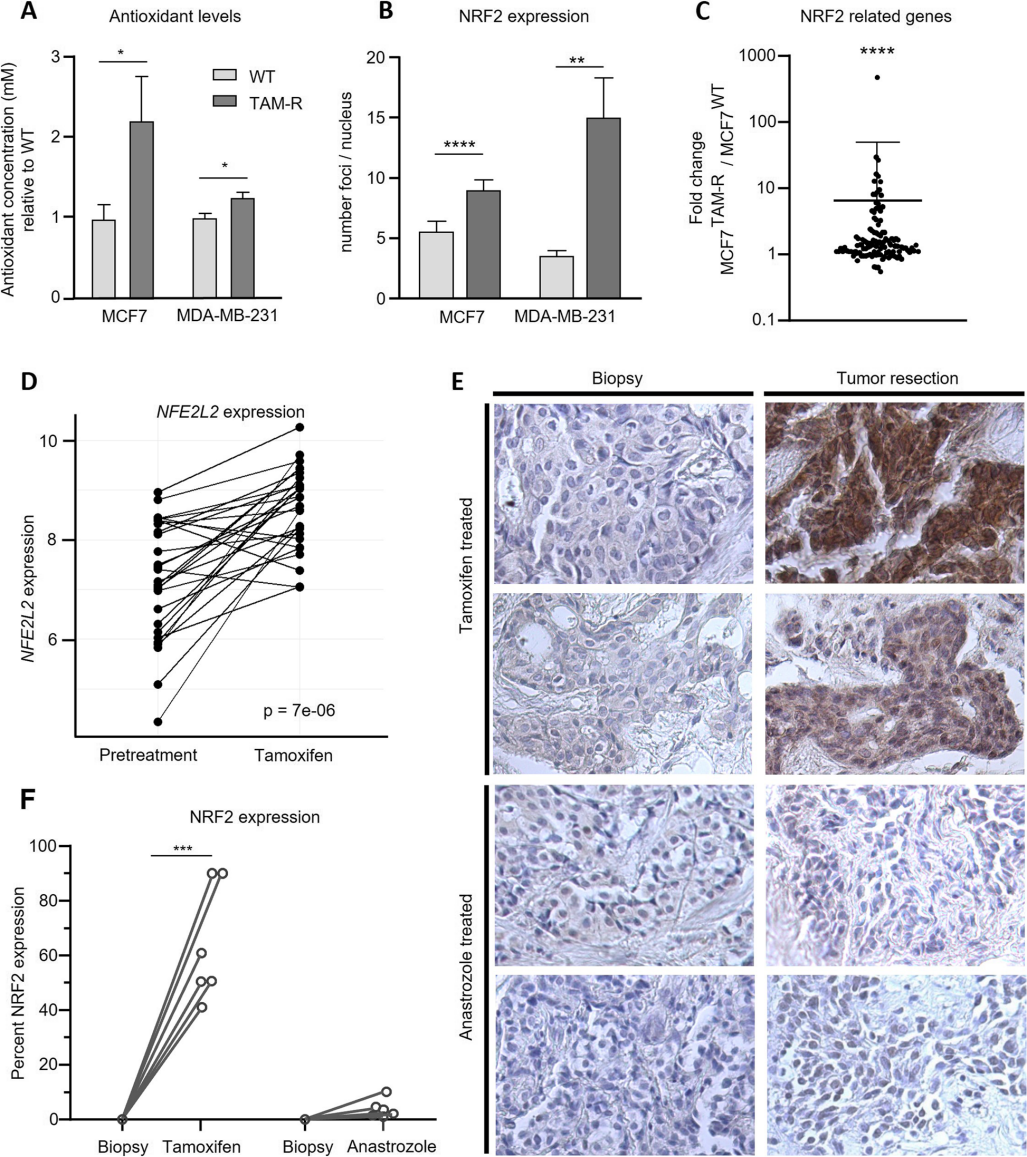
Antioxidant signaling is increased in TAM cells and antioxidant gene expression upregulated in patients after neoadjuvant tamoxifen. **A** Antioxidant capacity of WT and TAM-R cells (*n* = 6). **B** NRF2 expression of WT and TAM-R cells. **C** Fold change of NRF2 related genes in MCF7 cells. **D** Tumor gene expression of *NFE2L2* in 28 patients treated with neoadjuvant tamoxifen before and after tamoxifen treatment. **E** Histology of NRF2 expression on tumor material before (biopsy) and after (tumor resection) tamoxifen (*n* = 6) or anastrozole (*n* = 6) treatment. **F** NRF2 protein expression in patients treated with neoadjuvant tamoxifen or anastrozole before and after treatment (*n* = 6 for both groups). Data are represented as mean + SD (**A**). Statistical significance was determined by Student’s *t* test (**A**, **B**), one sample *t* test (**C**), and paired *t* test (**D**, **F**). *, *p* < 0.05; **,*p* < 0.01; ***, *p* < 0.001; ****, *p* < 0.0001

To directly evaluate cellular antioxidant capacity, we measured antioxidant capacity in MCF7 and MDA-MB-231 cells relative to a Trolox standard (6-hydroxy-2,5,7,8-tetramethylchroman-2-carboxylic acid, a strong antioxidant analog of vitamin E) and show significantly increased relative antioxidant levels in TAM-R cells compared to WT. Antioxidant capacity in MCF7^TAM−R^ was about twice as high as in MCF7^WT^ (*p* = 0.04; Fig. 4A), and also MDA-MB-231^TAM−R^ exhibited a significantly higher antioxidant concentration than MDA-MB-231^WT^ (*p* = 0.017).

We used immunofluorescent staining to verify increased nuclear NRF2 expression in vitro in MCF7 and MDA-MB-231 wild-type and tamoxifen-resistant cells. NRF2 is one of the major ROS-activated transcription factors stimulating antioxidant gene expression. In both cell lines, the tamoxifen-resistant cells showed a significantly upregulated number of NRF2 foci compared to wild-type cells (*p* < 0.0001 for MCF7, *p* = 0.009 for MDA-MB-231; Fig. 4B and Supplementary Figure S4).

Additionally, we reanalyzed gene expression data from previously established RNA sequencing of MCF7^WT^ and MCF7^TAM−R^ cells [6] for genes reportedly related to NRF2 and implicated in antioxidant signaling pathways (Supplementary Table S1). From the 110 genes examined, 53% were not altered in expression, whereas 17% exhibited a fold change between 1.4 and 2, and 26.4% of the genes showed an increased expression in the MCF7^TAM−R^ cells with a fold change higher than 2. In total, the analysis revealed a general statistically significant increase in genes related to NRF2-related antioxidant signaling in MCF7^TAM−R^ (*p* < 0.0001; Fig. 4C).

For clinical validation we evaluated tumor samples of breast cancer patients for an upregulation of NRF2 and *NFE2L2*, the gene encoding for NRF2, as reaction to tamoxifen treatment. Published expression profiles of patients treated with short-term presurgical tamoxifen were analyzed for *NFE2L2* gene expression (NCT00738777, [15]). Tumors from 28 breast cancer patients for whom paired data was available from before and after tamoxifen treatment were analyzed for *NFE2L2*, which was significantly increased after only 8–16 days of tamoxifen (*p* < 0.0001; Fig. 4D), showing that tumors quickly react to tamoxifen by upregulating antioxidant expression.

Tumor tissue was available from 6 out of the 28 patients originating from a biopsy before treatment and after short-term presurgical tamoxifen prior to surgical tumor excision. To rule out a general treatment effect leading to upregulation of *NFE2L2*, we also selected a set of paired samples from 6 patients treated with short-term presurgical anastrozole ± fulvestrant, other estrogen signaling inhibitors, from the same trial as control group. Tumor samples before and after treatment were stained for NRF2 to compare protein expression between groups. NRF2 expression was negative in all biopsies and strongly increased after tamoxifen treatment in all 6 patients ranging from 50 to 90% of tumor cells staining positive for NRF2 (*p* = 0.0008; Fig. 4E, F). Treatment with anastrozole ± fulvestrant, did not significantly increase NRF2 expression in breast tumor tissues (0–10% of tumor cells; Fig. 4E, F).

### Antioxidant inhibition re-sensitized tamoxifen-resistant cells to ROS and irradiation

**Fig. 5:**
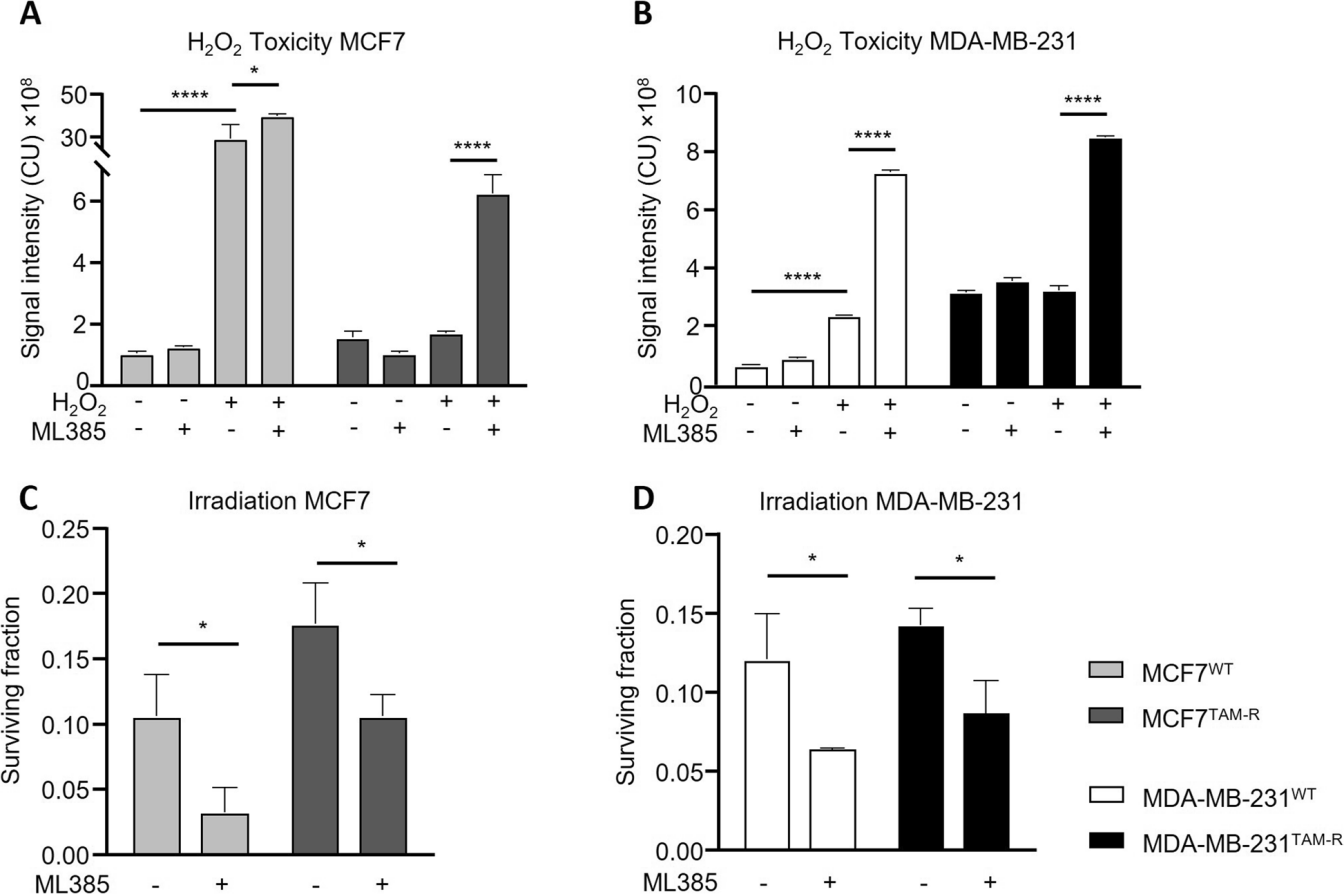
Response to H_2_O_2_ and irradiation after antioxidant inhibition with ML385. Toxicity of H_2_O_2_ with or without prior treatment with ML385 on WT and TAM-R (**A**) MCF7 and (**B**) MDA-MB-231 cells. Surviving fraction of (**C**) MCF7 and (**D**) MDA-MB-231 cells after irradiation with 4 Gy and prior treatment with or without ML385. Data are represented as mean + SD (*n* = 3). Statistical significance was determined by ANOVA with Sidak post hoc test for the area under the curve (**A**, **B**) and surviving fraction (**C**, **D**) comparing TAM-R cells to WT cells per condition. *, *p* < 0.05; ****, *p* < 0.0001

Decreased ROS levels and increased antioxidant capacity in TAM-R cells and patient tumor material suggest that long-term tamoxifen induces radioresistance through these mechanisms, thereby rendering cells resistant to ROS. To assess this, we inhibited antioxidant gene expression by NRF2 inhibition and measured toxicity of H_2_O_2_ in WT and TAM-R cells. ML385 treatment alone exhibited no toxic effects on cells. As shown before, H_2_O_2_ significantly increased toxicity in MCF7^WT^ (*p* < 0.0001), but not in MCF7^TAM−R^ (Fig. 5A). While ML385 further increased H_2_O_2_ toxicity only slightly in MCF7^WT^, H_2_O_2_ exhibited significantly higher toxicity on MCF7^TAM−R^ after prior ML385 treatment compared to PBS or H_2_O_2_ alone (*p* < 0.0001).

We show the same response for MDA-MB-231 cells whereas H_2_O_2_ alone exhibited a smaller toxic effect on MDA-MB-231^WT^ than on MCF7^WT^. However, toxicity was significantly increased in WT cells after H_2_O_2_ treatment (*p* < 0.0001), which was further increased with ML385 (*p* < 0.0001). Just as in MCF7^TAM−R^, H_2_O_2_ alone was not toxic on MDA-MB-231^TAM−R^, but prior treatment of ML385 significantly increased toxicity (*p* < 0.0001; Fig. 5B).

To assess re-sensitization to irradiation by NRF2 inhibition, cells were treated with ML385 prior to irradiation (4 Gy). As with direct ROS treatment, cells exhibited a significantly stronger response to irradiation with ML385 than without prior ML385 treatment (MCF7^WT^ *p* = 0.0109, MCF7^TAM−R^ *p* = 0.0122, Fig. 5C; MDA-MB-231^WT^ *p* = 0.0157, MDA-MB-231^TAM−R^ *p* = 0.0191, Fig. 5D). After NRF2 inhibition, MCF7^TAM−R^ exhibited a sensitivity to irradiation comparable to the natural radiosensitivity of MCF7^WT^.

## Discussion

Here, we established ERα-independent mechanisms underlying tamoxifen-induced radioresistance in breast cancer. After validating the cross-resistance of TAM-R cells to irradiation we reported earlier [6] in MCF7 and MDA-MB-231 cells, we observed increased mitochondrial ROS production upon tamoxifen treatment which was not displayed by TAM-R cells. The latter exhibited decreased ROS levels and sensitivity as well as increased glycolysis and antioxidant capacity, which is strongly related to decreased sensitivity to irradiation in vitro [10]. Heightened antioxidant capacity was accompanied by elevated NRF2 protein expression in TAM-R cells. Increased *NFE2L2*/NRF2 expression after short-term presurgical tamoxifen treatment (NCT00738777, [15]) clinically validates the increase in ROS in breast cancer patients. After longer-term treatment, this increase in antioxidant capacity of tumors may subsequently lead to decreased sensitivity to irradiation. Inhibition of antioxidant expression successfully re-sensitized cells to irradiation.

Our previous work on tamoxifen-induced radioresistance initially focused on ERα-positive breast cancer cells [6]. We hypothesized a connection with interferon signaling pathways based on RNAseq pathway analysis of tamoxifen-resistant and radiotherapy-resistant cells and in breast cancer tissue [6]. However, modulation of interferon signaling did not affect radiosensitivity (unpublished data). Considering that both tamoxifen and irradiation promote increased ROS signaling, the reported increase in IFN signaling is likely a result of a chronically heightened stress response due to chronic tamoxifen or irradiation, but unlikely to be the cause for tamoxifen-induced radioresistance given the results we present here.

Instead, we found that chronic tamoxifen treatment initiated metabolic adaptations as decreased OCR and SRC translating to decreased mitochondrial function. To further investigate whether mitochondrial dysfunction alone would lead to radioresistance, dysfunction was modelled by mitochondrial depletion. Rho0 cells were more radiosensitive than WT cells, indicating that mitochondrial dysfunction and resulting metabolic adaptations alone are not the single main cause for radioresistance. Literature is very heterogeneous on the matter of radiosensitivity in Rho0 cells. Human pancreatic Rho0 tumor cells were reported to be more radioresistant [9], while osteosarcoma and lung adenocarcinoma cells with the Rho0 phenotype displayed stronger radiosensitivity than the corresponding WT cells [21]. As most physiological ROS originate from the mitochondria, Rho0 cells have likely been exposed to significantly lower ROS throughout cell culturing. This could result in a naturally reduced antioxidant capacity rendering Rho0 cells more sensitive to irradiation-induced ROS.

In contrast to our results in MCF7 cells, we did not observe altered OXPHOS or glycolysis levels in the MDA-MB-231^TAM−R^ cells. However, MDA-MB-231 cells have been reported to generally rely more on glycolysis for basic cellular metabolism; while MCF7 cells use OXPHOS for 80–90% of their ATP production [22], MDA-MB-231 cells were shown to be less efficient in OXPHOS than MCF7 cells [23], to be highly glycolytic and have a preference for glycolysis [24]. This also supports our finding of higher glycolytic levels in MDA-MB-231^WT^ compared to MCF7^WT^ cells. Following, also in MDA-MB-231 increased antioxidant capacity plays a major role in tamoxifen-induced radioresistance although seemingly less dependent on glycolytic capacity.

Tamoxifen was long considered a general inhibitor of several OXPHOS complexes [11, 12], which related to decreased mitochondrial function reported after chronic tamoxifen treatment. However, as recently published, tamoxifen is unable to access intact mitochondria and can therefore not directly inhibit OXPHOS in living cells [25]. This corresponds to our finding that acute tamoxifen treatment did not influence the OCR in either MCF7^WT^ or MCF7^TAM−R^. Chronic mitochondrial dysfunction as observed in MCF7^TAM−R^ therefore likely takes long-term treatment leading to for instance incorporation into cellular membranes [26], Ca^2+^ overload, or other mechanisms described by Unten et al. [25]. On the other hand, acute tamoxifen treatment increased ROS production in WT cells, while also in breast cancer patients ROS signaling pathways have been shown to be upregulated after tamoxifen treatment compared to before treatment [15].

To investigate whether increased ROS production is a direct result of OXPHOS inhibition, we assessed ROS levels upon OXPHOS inhibition with IACS-010759 and Metformin. Both inhibitors significantly increased ROS levels in WT cells. However, as tamoxifen seems to be unable to penetrate into the mitochondria [25], it is unlikely to cause ROS through OXPHOS inhibition. Nevertheless, we show that mitochondrially depleted 67NR^Rho0^ cells display significantly lower ROS levels in reaction to tamoxifen treatment than 67NR^WT^ cells, which shows that tamoxifen-induced ROS largely originate from the mitochondria. This might be related to Ca^2+^ retention and accumulation in mitochondria, which has repeatedly been shown to be increased by tamoxifen [25, 27], and directly associated with ROS production [28]. The slight increase in ROS levels that we still observe in ROS levels in 67NR^Rho0^ cells after treatment with high tamoxifen concentrations might originate from NADPH oxidases, the second main producer of cellular ROS besides mitochondria, located in various cellular compartments [29]. In essence, ROS levels were significantly decreased in TAM-R cells compared to WT cells independent of treatment with tamoxifen, IACS-010759 or Metformin.

Also direct treatment with the ROS tert-butyl hydroperoxide did not increase ROS fluorescent signaling in TAM-R cells. Besides suggesting lower ROS production in TAM-R cells, these data indicate pronounced ROS-protective mechanisms in TAM-R cells, rendering cells resistant to ROS mediated damage and cell death. This interpretation is in line with previous findings in tamoxifen-resistant T47D breast cancer cells which were shown to have superior antioxidant capacities [30]. Therefore, it was assumed that these cells were able to reduce oxidative stress-mediated cell death. Indeed, we report increased total antioxidant capacity in TAM-R cells, supported by increased NRF2 expression in vitro and in patient tumor samples. This would inadvertently decrease the therapeutic effects of tamoxifen and other ROS-based therapies especially long term, when tumors adapt to persistently increased ROS by chronic upregulation of antioxidant expression.

As many cancer therapies, including irradiation and many chemotherapies, rely on ROS for tumor cell killing [31], chronically elevated antioxidant pathways would strongly diminish the cellular response to those therapies. An important electron donor for cellular antioxidant defense mechanisms is NADPH, which is produced in great amounts by glycolysis and following activation of the pentose phosphate pathway, also giving rise to other antioxidants as pyruvate and lactate. Both after acute and chronic tamoxifen treatment, we report increased glycolysis, likely as a cellular attempt to rapidly reduce tamoxifen-induced ROS. Through those increases in metabolically arising antioxidants, the more glycolytic phenotype which we report after chronic tamoxifen treatment has been related directly to radioresistance [10, 32].

Another major regulator of antioxidant gene expression is NRF2, the main activator of the antioxidant response element [33], which we show to be upregulated in MCF7^TAM−R^ cells compared to MCF7^WT^ cells. Besides, we report that genes related to the antioxidant signaling function of NRF2 were significantly upregulated in vitro. Regarding to literature, the best known mechanism of NRF2 activation is dependent on KEAP1 (Kelch-like ECH-associated protein 1) [34, 35]. KEAP1 continuously ubiquitinates NRF2, leading to NRF2 degradation by the proteasome. Upon ROS, KEAP1 gets inactivated through oxidative modification, leading to stabilization of NRF2. The activated NRF2 can then translocate to the nucleus to activate transcription of antioxidant proteins to increase resistance to oxidative stresses [34, 35]. As we show significantly increased ROS levels after tamoxifen treatment, it is likely that the observed subsequent activation of NRF2 is dependent on KEAP1.

Previously, high NRF2 expression in breast cancer patients at the time of diagnosis has been linked to poorer survival after tamoxifen therapy [13] and has been shown to be key to chemotherapy resistance in MCF7 cells [36]. In other tumor types, high NRF2 expression has also been linked to reduced benefit from cisplatin/vinorelbine chemotherapy [37] and chemoradiation therapy [38] in lung and esophageal squamous cell carcinoma respectively. Using tumor material from patients treated with short-term presurgical tamoxifen, we clinically validated the increased antioxidant capacity found in vitro by analyzing tumor *NFE2L2* gene expression and staining for NRF2, comparing expression before and after treatment. Upregulation of *NFE2L2* gene expression as well as NRF2 protein expression after tamoxifen, but not after treatment with other estrogen pathway modulators confirms that clinically used concentrations of tamoxifen indeed increased ROS levels and activate NRF2 expression to increase antioxidant gene expression in patients, similar to what we observed in vitro. It is important to note that, in this study, patients received short-term tamoxifen prior to surgery leading to a maximal treatment period of 16 days. Neoadjuvant treatment is given for 4–8 months, while in an adjuvant setting, patients are treated with tamoxifen for a period of 5 years or longer.

Considering that we show a rapid response of tumors to tamoxifen by upregulation of an antioxidant response in vitro and in tumors of patients, it is to be expected that tumor cells would adapt to chronic tamoxifen-induced ROS exposure. This would lead to chronically increased antioxidant expression by which tumors may escape the anti-tumor effects of not only tamoxifen but other ROS-mediated therapies as well. In the situation of tumor recurrence, treatment options would be drastically reduced as tumors have developed resistance to tamoxifen as well as radiotherapy. Prospectively, drugs are under development with the aim to reduce NRF2 expression and re-sensitize tumors to ROS-dependent therapies [39].

As elevated antioxidant expression seems to be the main reason for tamoxifen-induced radioresistance, we attempted to inhibit this antioxidant overexpression. Inhibition of NRF2 with ML385 re-sensitized cells to H_2_O_2_ and irradiation, showing that cross-resistance can be averted by inhibiting NRF2 as the main activator of antioxidant expression. ML385 directly interacts with the NRF2 protein to inhibit transcriptional activity and has previously been reported to enhance the efficacy of chemotherapeutic drugs in lung cancer cells [40]. ML385 has not been clinically tested and clinical inhibitors developed for specific NRF2 inhibition are currently lacking. However, Omacetaxine mepesuccinate (Synribo™), a plant alkaloid approved for chronic myeloid leukemia [41] and effective in triple-negative breast cancer [42], has recently been reported to act as a chemical inhibitor of NRF2 [43]. The drug was shown to suppress NRF2 dependent antioxidant gene expression in lung carcinoma cells. It remains to be evaluated if Omacetaxine mepesuccinate may be useful in tamoxifen-resistant breast cancer and could possibly re-sensitize cells to ROS-dependent therapies. Yet, considering that the drug has shown clinically meaningful responses in patients with multiple resistances to tyrosine kinase inhibitor chemotherapies [44], which has been related to NRF2 pathway disruption [45], Omacetaxine mepesuccinate seems promising for possible treatment of patients with tamoxifen-resistant breast cancer. Nonetheless, a drug specific to NRF2 would be preferential, as non-specific effects of Omacetaxine mepesuccinate as for instance inducer of cell cycle arrest [46] remain.

## Conclusions

This study highlights the importance of cellular metabolism and anti-oxidant capacity regarding sensitivity to radiotherapy. We show that tamoxifen-induced, mitochondrial ROS lead to a rapid antioxidant response in vitro as well as in tumors of breast cancer patients that were treated with tamoxifen. Chronic tamoxifen treatment leading to persistently high ROS levels brings about cellular adaptations of strongly elevated antioxidant capacity dependent on NRF2, to which glycolysis is likely a large contributor. This evokes a general cellular resistance to ROS, which diminishes the sensitivity to ROS-mediated therapies such as irradiation. Radioresistance induced by tamoxifen treatment could be reversed by reducing antioxidant gene expression through pharmacological NRF2 inhibition.

### Supplementary Information

**Additional file 1: Figure S1.** No difference in OCR or ECAR was detected between the WT and TAM-R cells or with acute tamoxifen treatment.

**Additional file 2: Figure S2.** Mitochondrial depletion increases cellular sensitivity to irradiation.

**Additional file 3: Figure S3.** 67NRWT cells displayed significantly increased ROS levels after treatment with tamoxifen already after treatment with 1 μM of tamoxifen (*p* = 0.0001) which dose-dependently increased up to 10 μM tamoxifen

**Additional file 4: Figure S4.** The tamoxifen-resistant cells showed a significantly upregulated number of NRF2 foci compared to wild-type cells.

**Additional file 5: Table S1.** Gene expression data from previously established RNA sequencing of MCF7WT and MCF7TAM-R cells [6] for genes reportedly related to NRF2 and implicated in antioxidant signaling pathways

## Data Availability

The datasets analysed during the current study are available in the GEO repository, Platform ID: GPL28292, Series GSE147271 [https://www.ncbi.nlm.nih.gov/geo/query/acc.cgi?acc=GSE147271]. The datasets generated and analyzed during the current study are available from the corresponding author on reasonable request.

## Abbreviations

TAM-R: Tamoxifen resistant
WT: Wildtype
ROS: Reactive oxygen species
ERα: Estrogen receptor alpha
OXPHOS: Oxidative phosphorylation
OCR: Oxygen consumption rate
ECAR: Extracellular acidification rate
SRC: Spare respiratory capacity
