# Supplementary figures and images for: Tamoxifen induces radioresistance through NRF2-mediated metabolic reprogramming in breast cancer
Source: Cancer Metab. 2023 Feb 8;11:3. doi: 10.1186/s40170-023-00304-4 (PMC9909892; doi:10.1186/s40170-023-00304-4)

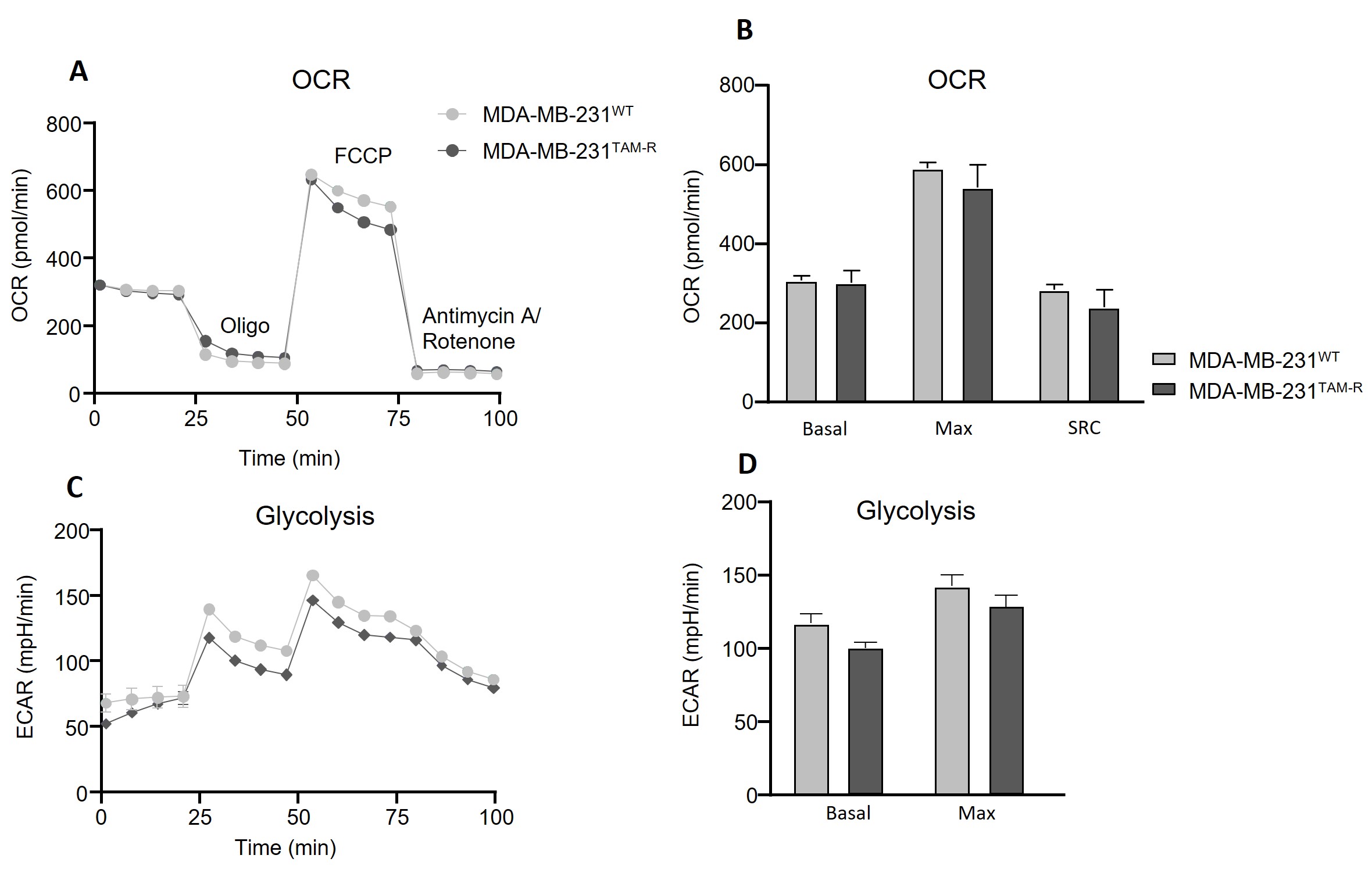

Supplement: Supplementary file 2 — Additional file 2: Figure S2. Mitochondrial depletion increases cellular sensitivity to irradiation. [file 40170_2023_304_MOESM2_ESM.jpg]

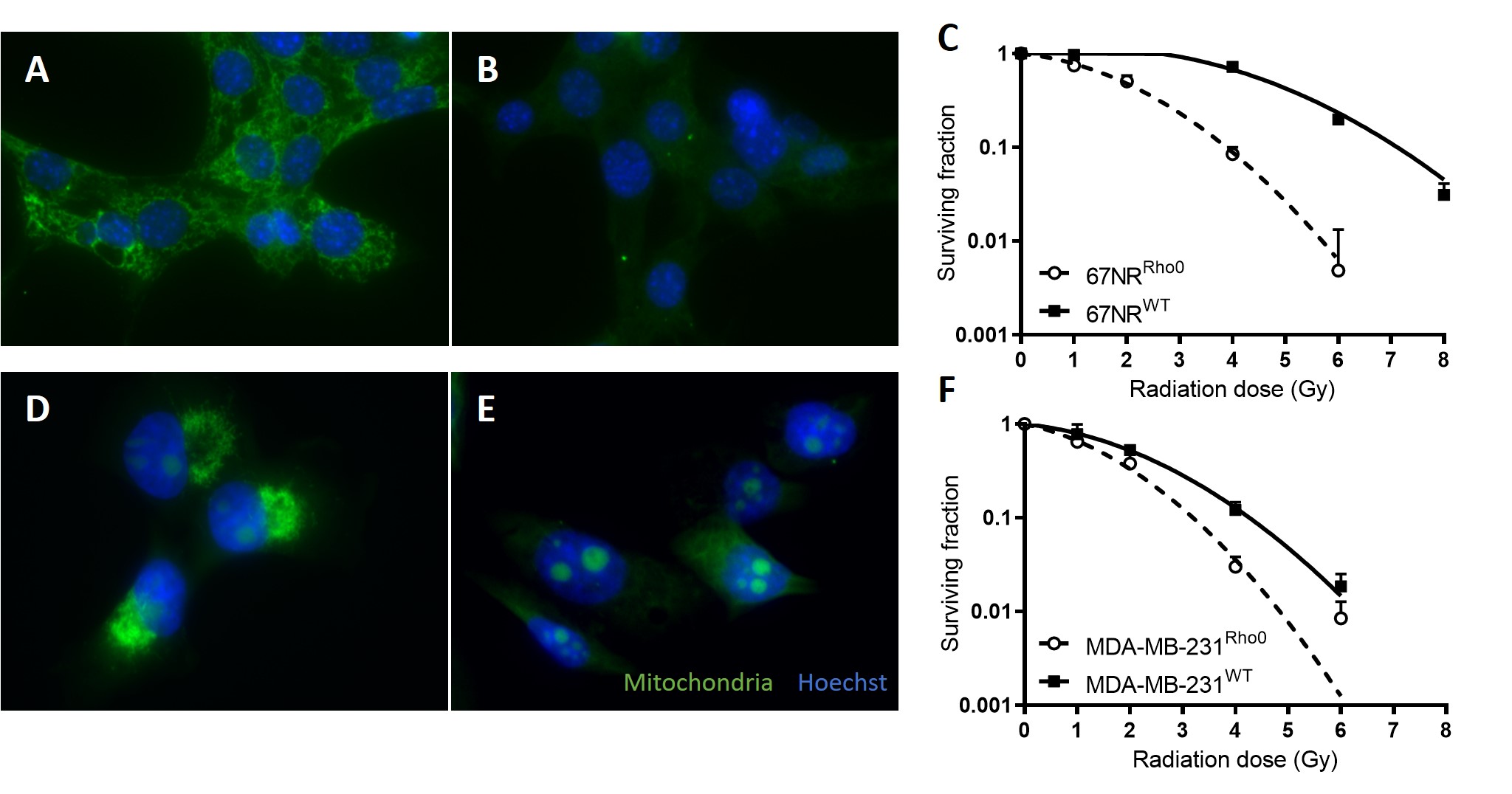

Supplement: Supplementary file 3 — Additional file 3: Figure S3. 67NRWT cells displayed significantly increased ROS levels after treatment with tamoxifen already after treatment with 1 μM of tamoxifen (p = 0.0001) which dose-dependently increased up to 10 μM tamoxifen [file 40170_2023_304_MOESM3_ESM.jpg]

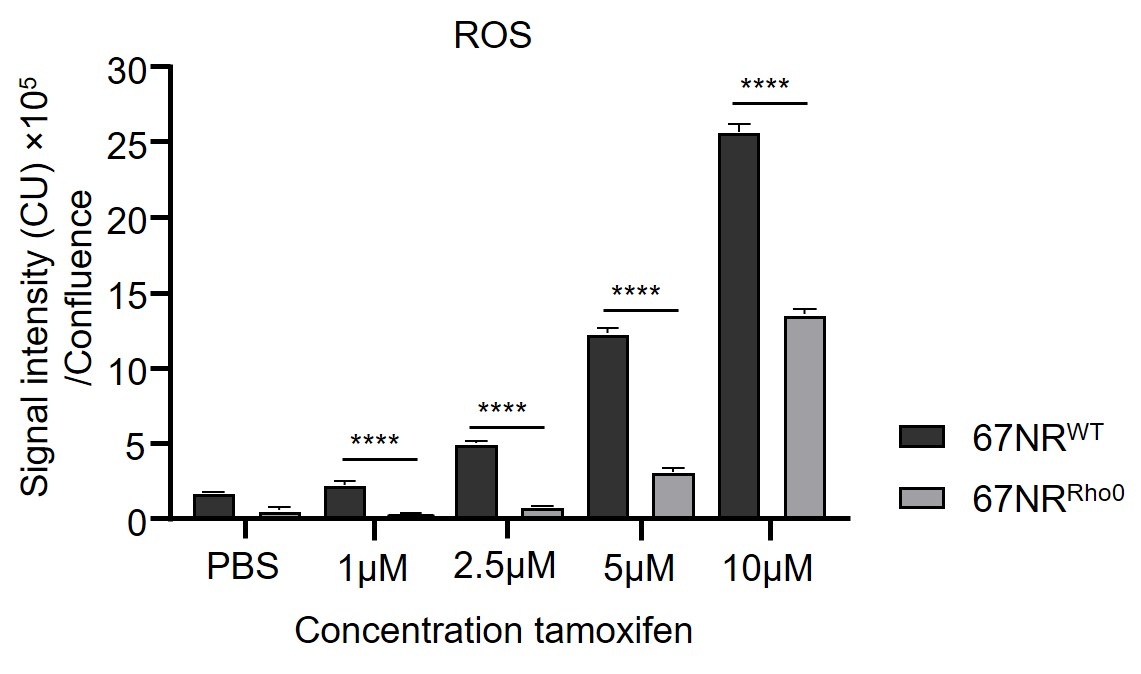

Supplement: Supplementary file 4 — Additional file 4: Figure S4. The tamoxifen-resistant cells showed a significantly upregulated number of NRF2 foci compared to wild-type cells. [file 40170_2023_304_MOESM4_ESM.jpg]

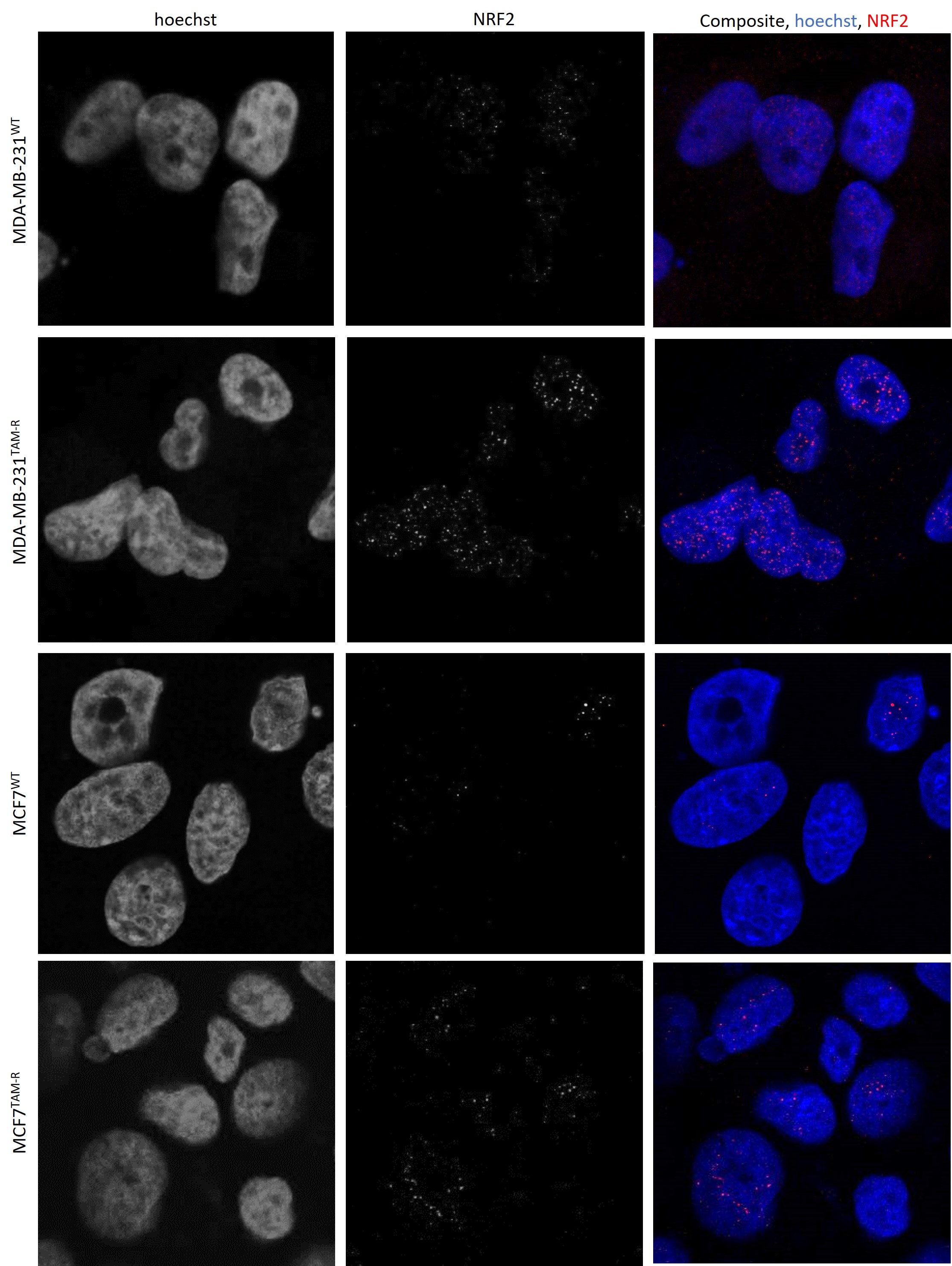

Supplement: Supplementary file 5 — Additional file 5: Table S1. Gene expression data from previously established RNA sequencing of MCF7WT and MCF7TAM-R cells [6] for genes reportedly related to NRF2 and implicated in antioxidant signaling pathways [file 40170_2023_304_MOESM5_ESM.jpg]
